# Shift in tuna catches due to ocean warming

**DOI:** 10.1371/journal.pone.0178196

**Published:** 2017-06-07

**Authors:** Alberto Monllor-Hurtado, Maria Grazia Pennino, José Luis Sanchez-Lizaso

**Affiliations:** 1 Department of Marine Science and Applied Biology, University of Alicante, Alicante, Spain; 2 Fishing Ecology, Management and Economics, Federal University of Rio Grande do Norte (UFRN), Campus Universitário s/n, Natal, Brasil; 3 Instituto Español de Oceanografía, Centro Oceanográfico de Murcia. C/ Varadero 1, San Pedro del Pinatar. Murcia. Spain; 4 Statistical Modeling Ecology Group (SMEG). Departament d'Estadística i Investigació Operativa, Universitat de València. C/Dr. Moliner 50, Burjassot, Valencia, Spain; University of Tasmania, AUSTRALIA

## Abstract

Ocean warming is already affecting global fisheries with an increasing dominance of catches of warmer water species at higher latitudes and lower catches of tropical and subtropical species in the tropics. Tuna distributions are highly conditioned by sea temperature, for this reason and their worldwide distribution, their populations may be a good indicator of the effect of climate change on global fisheries. This study shows the shift of tuna catches in subtropical latitudes on a global scale. From 1965 to 2011, the percentage of tropical tuna in longliner catches exhibited a significantly increasing trend in a study area that included subtropical regions of the Atlantic and western Pacific Oceans and partially the Indian Ocean. This may indicate a movement of tropical tuna populations toward the poles in response to ocean warming. Such an increase in the proportion of tropical tuna in the catches does not seem to be due to a shift of the target species, since the trends in Atlantic and Indian Oceans of tropical tuna catches are decreasing. Our results indicate that as populations shift towards higher latitudes the catches of these tropical species did not increase. Thus, at least in the Atlantic and Indian Oceans, tropical tuna catches have reduced in tropical areas.

## Introduction

Anthropogenic impact on marine ecosystems is widely distributed all over the world. Climate change and fishing activity are considered to have the most widespread impact on marine ecosystems [1], affecting temperature, salinity, wind fields, oxygen, pH, and the density structure of the water column [2]. In the upper 75 m of the ocean, the global average warming trend has been 0.11°C per decade over the period 1971–2010 [3].

In response to ocean warming, marine populations tend to move toward the poles and deeper depths [4–7]. This is reflected as increases in warmer water species in some higher latitude areas [8–10] and leads to the appearance of invasive species in other locations [11,12]. Shifts in the ecology and biogeography of marine fishes may be useful indicators of climate changes [13]. Due to the wide distribution of tuna species [14] and their dependence on optimal temperature, their populations may be a good indicator of the effect of climate change on global fisheries.

Tuna and tuna-like fishes include approximately forty species living in the Atlantic, Indian and Pacific Oceans and the Mediterranean Sea. They are very important economically, mainly as a significant human food source. The annual catch of these fishes has tended to increase continually from less than 0.6 million tonnes in 1950 to more than 6 million tonnes [15].

The Thunnini are distinguished among all bony fishes by the counter-current heat exchanger system (rete mirabile) [16] that maintains their body temperatures above that of the ambient water [17]. These fish are constantly swimming to counterbalance their negative buoyancy and moving extensively in search of food for energy. This strategy is aided by other physiological and morphological adaptations for thermoregulation and high-efficiency oxygen extraction [17]. Despite these adaptations, sea temperature is an important environmental parameter for tuna distribution [18].

A distinction is made between tropical and temperate tunas, since they show different distributions due to their specific thermal tolerances and are caught by different fisheries. Tropical tunas are found in waters with temperatures higher than 18°C (although they can dive to colder waters), whereas temperate tuna are found in waters as cold as 10°C or colder, but can also be found in tropical waters [14, 18].

Approximately 66% of total global tuna catch is composed of a few species: skipjack tuna (*Katsuwonus pelamis*) 58.1%, yellowfin tuna (*Thunnus albacares*) 26.8%, bigeye tuna (*Thunnus obesus*) 8.2%, albacore (*Thunnus alalunga*) 5.9%, Atlantic bluefin tuna (*Thunnus thynnus*) <1% and southern bluefin tuna (*Thunnus maccoyii*) (<1%) [15]. According to their thermal range and distribution [14, 18], these major species can be classified into tropical (skipjack and yellowfin), intermediate (bigeye) and temperate tunas (albacore, Atlantic bluefin and southern bluefin).

This study analyzed trends in sea temperature (SST) and tropical tuna percentage in longliner catches in the Atlantic, Western Pacific and Indian Oceans during the period 1965–2011 (1967–2011 in the Indian Ocean) to evaluate the effect of ocean warming on spatial distribution of tropical tuna over the past decades. We hypothesize that in subtropical areas the proportion of tropical tuna in the catches will increase as SST increases.

## Material and methods

Longliner catch and effort data were obtained from the International Commission for the Conservation of the Atlantic Tuna (ICCAT-database CATDIS and T2CE), the Western and Central Pacific Fisheries Commission (WCPFC) and the Indian Ocean Tuna Commission (IOTC). Sea surface temperature (SST) data were from the NOAA database, Extended Reconstructed Sea Surface Temperature V3b (ERSST).

All data were converted to annual 5x5° values by averaging. The IOTC and ICCAT data were in a variety of formats: 1x1, 5x5, 5x10, 10x20 and 20x20. Only 1x1 and 5x5 data were used in this analysis. 1x1 data were converted to 5x5. WCPFC datasets were already in a 5x5 format. All data were converted to annual values by averaging (ICCAT original values were recorded in trimesters, WCPFC in months and IOTC from 1 to several months). We thus obtained our dataset in 5x5° latitude/longitude grid squares starting from the coordinate 0/0°. Effort data are expressed as number of hooks per grid cell.

The analysis focused on the major tuna species: skipjack, yellowfin, bigeye, albacore and Atlantic bluefin tuna in the Atlantic; yellowfin, albacore and bigeye in the western Pacific; and skipjack, yellowfin, bigeye, albacore and southern bluefin tuna in the Indian Ocean. They were classified according to their thermal range and distribution [14, 18] in tropical (skipjack and yellowfin), intermediate (bigeye) and temperate tunas (albacore, Atlantic bluefin and southern bluefin). The percentage of tropical species (skipjack and yellowfin) was calculated for each grid cell.

SST data, originally in 2x2 monthly format, were converted to annual 5x5 SST by weighted average in order to unify the spatial resolution of all the data (both tuna and SST data). Only those grid cells that contained SST and catch data for at least 35 years in the period 1965–2011 were included in the analysis.

The data set used for the analysis is included as supplementary material (S1 and S2 Files).

### Modeling tropical tuna percentage

Tropical tuna percentage in longliner catch (%trop) can have any continuous value ranging between 0 and 100. This kind of data has frequently been modeled by transforming the dependent variable using the arcsine square root transformation [19, 20]. However, the approach has several drawbacks and inferences can be misleading. Indeed, model parameters cannot be easily interpreted in terms of the original response, and the measures of proportions typically display asymmetry [21]. Unfortunately, the symmetry of the normal distribution can result in nonsensical predictions, which means that confidence intervals are outside the [0, 1] range. The beta distribution contrasts with this and is very flexible in terms of shape and fulfils the required characteristics [22].

The %trop was modeled using a Bayesian beta regression model. Specifically, %trop, Y_i_ in each grid cell *j*, was assumed to follow a beta distribution Y_j_ ~ Be(μ_j_, ȹ_j_). The observation year, the SST variable and the interaction between the latitude and the year were implemented as explanatory variables. In particular, the latter variable was implemented to test the direction of %trop distribution over time as the hypothesis of a poleward shift. For each ocean, models were computed for the northern and southern hemisphere separately. SST was treated as a continuous variable and the year as a factor.

Bayesian parameter estimates in the form of marginal posterior distributions were obtained throughout the R-INLA approach and software [23, 24].

Vague zero-mean Gaussian prior distributions with a variance of 100 were assigned for the fixed effects, as recommended by Held et al. (2010) [25]. These priors are approximations of non-informative priors designed to have little influence on posterior distribution.

Models were performed for each ocean and models were selected using the Watanabe Akaike Information Criterion (WAIC) [26], which is inversely proportional to the goodness of fit.

Spatial maps were then obtained applying a Bayesian kriging, aggregated per decade to remove effects of sub-decade variability. In order to better visualize the long term changes in the %trop, the difference between the first and last decades are shown on the maps.

Besides Bayesian analysis, a smoothing function was used to attempt to capture the general patterns in the time trends of %trop, effort, total catches and SST, while also reducing the noise. This technique is especially useful to visually assess the relationship between variables for long time series, where trends can be hard to visualize. Specifically, we smoothed the time series using locally weighted scatterplot smoothing (lowess), an outlier-resistant method that estimates a polynomial regression curve using local fitting [27].

In addition, we applied a bootstrap technique to each time series with the percentile method, in order to account for the variability in the original lowess fit. With this methodology, each series would have a 95% confidence interval for the original lowess [28, 29]. This technique was performed with the entire dataset, and then only for subtropical regions (20-30N, 20-30S) to explore if the effects of ocean warming are more evident in these regions than the others.

## Results

The final Bayesian models for all the oceans included all the explanatory variables, including the SST, latitude, year and interaction between latitude and year (Table 1).

**Table 1:** Watanabe Akaike Information Criterion (WAIC) comparison for all the models tested. The best model is highlighted in bold. Predictor acronyms are: SST = Sea temperature, Y = year, Lat = Latitude, YL = interaction between year and latitude.

	Model	Atlantic NorthH	Pacific NorthH	Indian NorthH	Atlantic SouthH	Pacific SouthH	Indian SouthH
1	1 + Y * Lat + SST	531	621	448	543	598	465
2	1 + Y + Lat +SST	548	628	457	559	605	472
3	1 + SST+ Lat	567	629	462	564	610	476
4	1 + Y + SST	571	632	467	567	615	478
5	1 + Y * Lat	578	637	471	572	618	480
6	1 + SST	573	640	470	573	620	481
7	1 + Lat	598	642	479	584	6623	484
8	1 + Y	601	648	486	585	625	482
9	1	624	653	489	588	630	486

Specifically, the SST had a positive effect on the expected percentage of tropical tuna in all the oceans, for both the northern and southern hemispheres. In the Atlantic, results showed that for a one degree rise in SST the proportion of tropical tuna is expected to increase by 1.46 in the northern hemisphere and by 1.32 in the southern (Table 2). Similarly, in the Pacific, the increase was 1.34 in the northern hemisphere and 1.27 in the southern. The Indian Ocean showed the lowest increase, 1.25 in the northern hemisphere and 1.19 in the southern.

**Table 2:** Numerical summary of the marginal posterior distribution for model parameters provided by the selected model for each case considered. For each variable the median (Q_0.5_), and a 95% credible central interval is provided, containing 95% of the probability under the posterior distribution.

Model	Variable	Q_0.5_	Q_0.025_	Q_0.975_
Atlantic NorthH	SST	1.46	1.15	2.36
	Year	1.28	1.12	1.71
	Lat	-1.42	-1.09	-2.05
	Y *Lat	1.38	1.10	1.68
Pacific NorthH	SST	1.34	1.12	1.72
	Year	1.31	1.03	1.86
	Lat	-1.72	-1.08	-2.20
	Y *Lat	1.26	1.12	1.73
Indian NorthH	SST	1.25	1.08	1.82
	Year	-1.19	-1.03	-1.53
	Lat	-1.20	-1.08	-1.68
	Y *Lat	1.19	1.09	1.70
Atlantic SouthH	SST	1.32	1.15	1.95
	Year	1.25	1.09	1.45
	Lat	1.21	1.08	1.65
	Y *Lat	-1.30	-1.07	-1.70
Pacific SouthH	SST	1.27	1.14	2.01
	Year	1.13	1.02	1.43
	Lat	1.75	1.05	2.32
	Y *Lat	-1.27	-1.08	-1.57
Indian SouthH	SST	1.20	1.09	1.75
	Year	1.15	1.06	1.32
	Lat	1.14	1.03	1.28
	Y *Lat	1.16	1.02	1.37

The interaction of year and latitude showed a positive effect in the three oceans in the northern hemisphere, highlighting a constant shift of the tropical species towards higher latitudes. Similarly, in the southern hemisphere the negative relationship indicates the same patterns, except for the Indian Ocean (Table 2).

The year and latitude themselves were also important. In particular, latitude showed a negative relationship in the northern hemisphere, against a positive pattern in the southern since negative values were used for southern hemisphere latitudes. The only exception was the positive relationship of the Indian Ocean in the southern hemisphere. A positive relationship for year appeared for almost all ocean models, although for the Indian the pattern was different in the northern hemisphere (Table 2).

**Fig 1:**
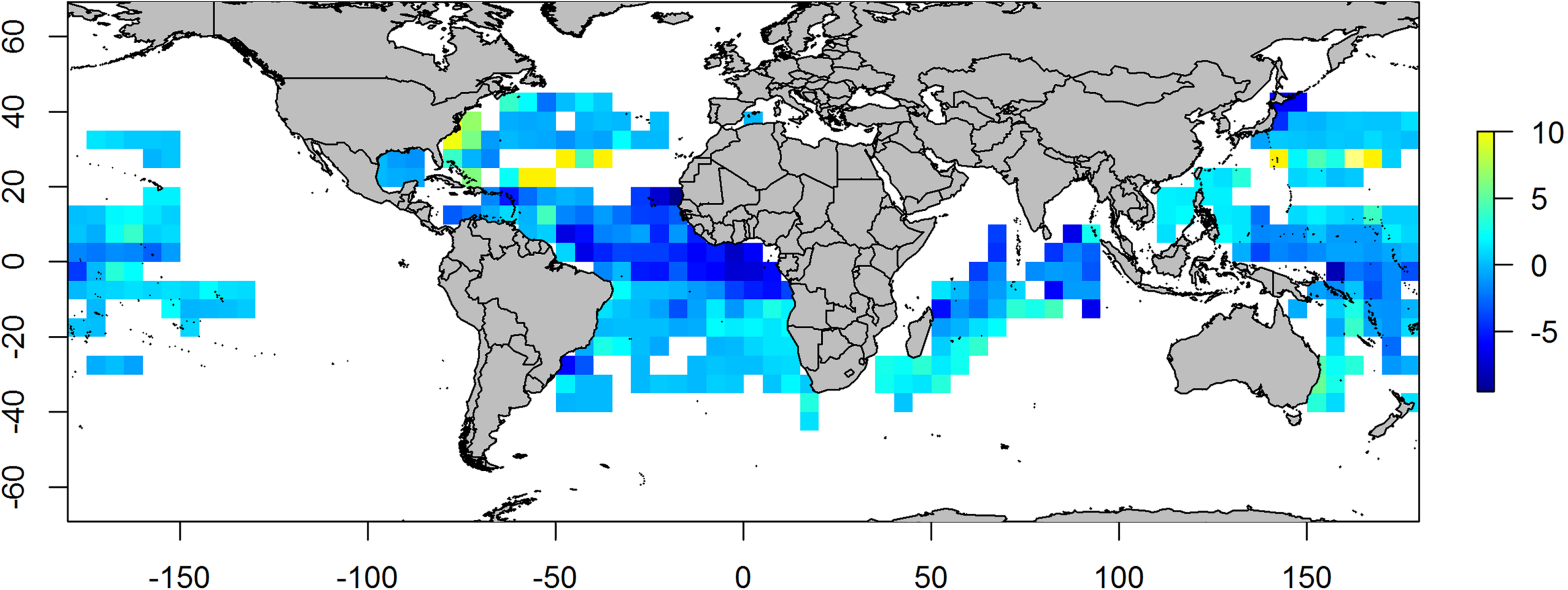
Map of the percentage of tropical tuna in longliner catches (%trop) changes from the first to the last decade of the time series 1967–2011.

Fig 1 shows the change in %trop from the first to the last decade of the time series, highlighting a stronger increasing pattern in the proportion of tropical tuna, especially in the subtropical areas (Fig 1). The decrease in tropical species in tropical areas is due to an increase of the contribution of bigeye tuna, particularly from the 1970’s onwards.

**Fig 2:**
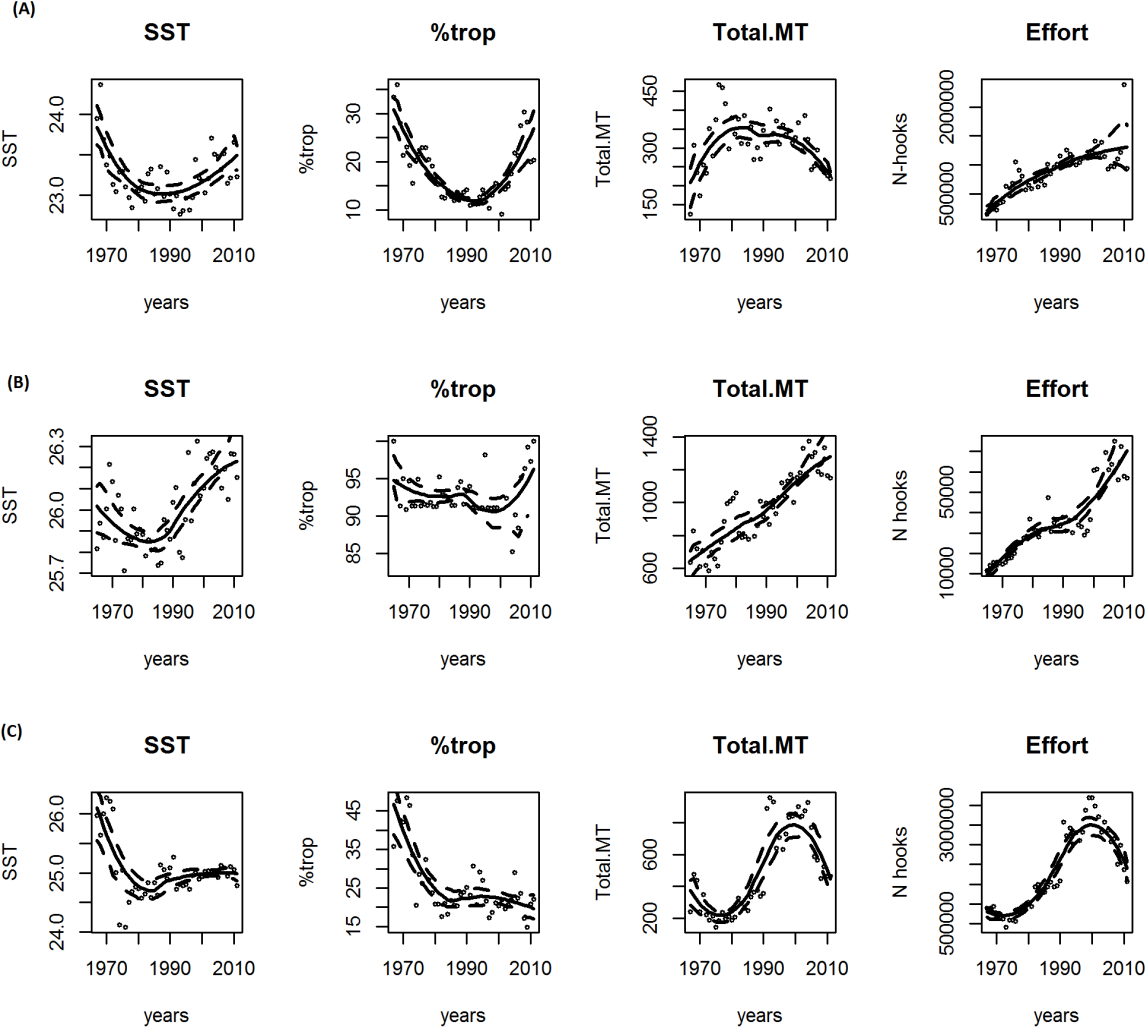
**Smooth functions for sea surface temperature (SST), tropical tuna percentage in longliner catches (%trop), total catches and effort for the entire time series (1965–2011) for the three oceans: (A) Atlantic; (B) Pacific; (C) Indian.** The solid line in each plot is the estimated smooth function and the dashed lines represent approximate 95% confidence intervals.

**Fig 3:**
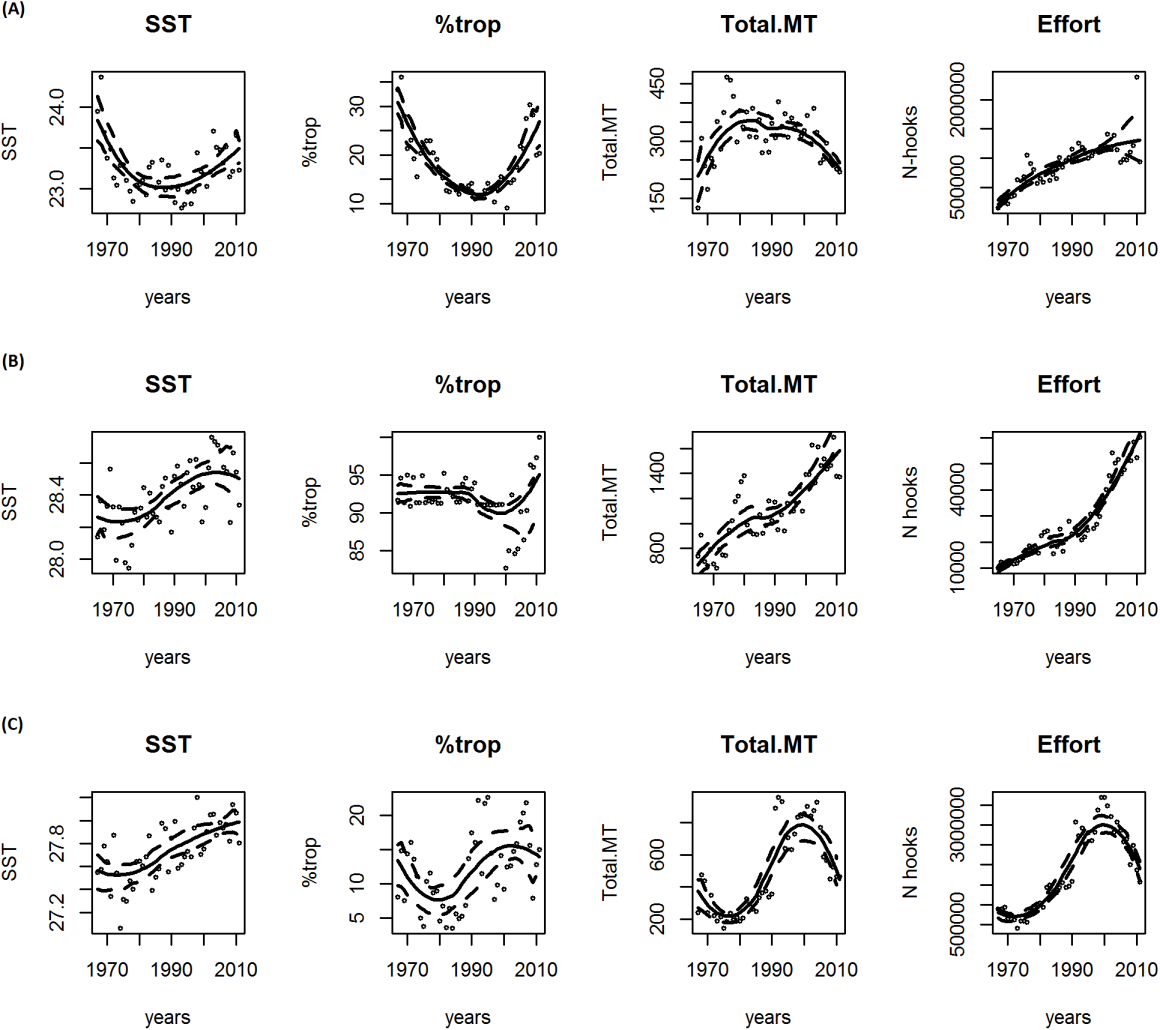
**Smooth functions for the sea surface temperature SST, tropical tuna percentage in longliner catches (%trop), the total catches and effort for the entire time series 1965–2011 for the three oceans in sub-tropical regions: (A) Atlantic; (B) Pacific; (C) Indian.** The solid line in each plot is the estimated smooth function and the dashed lines represent approximate 95% confidence intervals.

The bootstrap-smoothing graphic solution showed an increasing SST tendency in the Atlantic and Pacific oceans in the last two decades (Fig 2). This pattern is clearer when we restrict the analysis to subtropical areas (Fig 3). In contrast, the Indian Ocean showed a more stable pattern in the last two decades when analyzed on a large scale (Fig 2), while a clearly increasing tendency is seen in the subtropical areas (Fig 3).

The percentage of tropical tuna did not show a clear pattern when overall data were analyzed (Fig 2). In particular, the %trop tended to increase in the Atlantic and Pacific oceans, and decrease in the Indian ocean (Fig 2). However, the Atlantic and Pacific oceans showed an increasing trend when the analyses were restricted to subtropical areas (Fig 3). However, for the Indian Ocean %trop tends to decrease (Fig 3).

Catches showed an increasing trend only in the Pacific, both overall and in the subtropical areas, while a decreasing trend was found for the Atlantic and Indian oceans during recent decades (Fig 2 and Fig 3).

The effort showed an increasing trend in the Atlantic and Pacific, on both regional and local scales, whereas the Indian ocean stood out with a decreasing tendency using both approaches (Fig 2 and Fig 3).

## Discussion

Our results revealed a rising trend in SST and %trop in subtropical areas throughout the Atlantic and Pacific Oceans during the past four decades, and an even stronger increase in the last two decades. In addition, tropical tuna catches show a descending trend in the Atlantic, which may be taken as an indicator that the increased %trop was not related to a change in fishing strategy, at least in this ocean. This suggests that tuna populations at intermediate latitudes (20-30N and 20-30S) underwent a large-scale tropicalization from 1965 to 2011, in accordance with the findings of Cheung et al. (2013) [19]. That study showed that ocean warming has already been affecting global fisheries, resulting in an increasing dominance of catches of warmer water species at higher latitudes during the past four decades [19].

In the Pacific, the clear increase in %trop along the time series is not related to a trend towards decreasing catches. The effort and total catch increase together with %trop, highlighting a possible change in the fisheries strategy or a spatial displacement of the fishery to higher latitudes. This last hypothesis is supported by Bayesian analysis results that identify a constant spatio-temporal trend to higher latitudes.

The Indian Ocean is the only area in which the %trop pattern is not totally clear. Indeed, for subtropical areas the increasing %trop pattern showed the opposite trend in the last five years of the time series. However, Bayesian analysis indicates that the %trop is positively related with SST. Furthermore, similarly to the Atlantic Ocean, tropical tuna catches showed a decreasing pattern, as did the fishing effort. Neither was the spatio-temporal effect clearly identifiable in the Bayesian analysis, since the interaction of the year and latitude showed a positive relationship in both northern and southern hemispheres. Further analysis using data from recent years (2011–2017) should better define this pattern.

However, distributions of North Sea and Northeast Atlantic fishes responded markedly to ocean warming, shifting in intermediate latitudes [4, 5]. This phenomenon seems to be more important in species with short life cycles and small body size [4]. Tropical tunas are characterized by small to medium size, rapid growth, early age-at-maturity, long spawning duration and short life span. These species therefore display a rapid turnover, characteristic of *r*-selected species [20]. The tropicalization of longliner tuna catches in intermediate latitudes may be due to a shift of tropical tuna populations towards the poles in response to ocean warming. This tendency appears to be particularly strong in the Atlantic Ocean.

If this trend towards ocean warming continues [3], the prospects point to large scale redistribution in fishery catches in the near future [21, 22]. This would affect the most vulnerable economies in the world. Although warming will be most pronounced at high latitudes, countries most vulnerable to warming-related effects on fisheries lie in the tropics [23]. At least in the Atlantic and Indian, our results indicate that tropical tuna catches have reduced in tropical areas, as populations shift towards higher latitudes, since the expansion towards the poles of these tropical species did not increase their catches.

Finally, two main limitations have to be taken into account in this worldwide long series approach. Firstly, stock trends, catch regulations and market demands could also have significant effects on species targeting and catches. Consequently, SST may be only a partial driver of the trends identified in the different oceans. However, our analyses have focused on the proportion of tropical tunas and not on the total catch of the species, in order to avoid these external biases. Moreover, data are analyzed on a yearly time scale and seasonal patterns can be lost in such an approach. However, the aim of this study was to provide a first worldwide long-term series assessment of tropical tuna populations, so yearly data were the most useful.

## Supporting information

S1 File Reduced dataset including only cells with effort available. (XLSX)

S2 File Dataset including catches per species, year and cell. (XLSX)
